# Bibliometric and visualized analysis of scientific publications on subglottic stenosis based on web of science core collection

**DOI:** 10.1186/s13019-024-02515-2

**Published:** 2024-02-04

**Authors:** Yaping Zhang, Zhanqiu Dai, Qixin Xia, Yufeng Wu, TingxiaoZhao, Qi Chen, Chen Xia, Jun Zhang, Jiongnan Xu

**Affiliations:** 1 Center for General Practice Medicine, Zhejiang Provincial People’s Hospital (Affiliated People’s Hospital, Hangzhou Medical College), Hangzhou, Zhejiang 310014 China; 2 Center for Plastic & Reconstructive Surgery, Department of Orthopedics, Zhejiang Provincial People’s Hospital (Affiliated People’s Hospital, Hangzhou Medical College), Hangzhou, People’s Republic of China; 3 Bengbu Medical College, Bengbu, People’s Republic of China; 4 Hangzhou Medical College, Hangzhou, People’s Republic of China; 5 The Second School of Clinical Medicine, Zhejiang Chinese Medical University, Hangzhou, China; 6 Department of Orthopedics, Zhejiang Provincial People’s Hospital Bijie Hospital, Bijie, Guizhou China; 7 Department of Orthopaedics, The Second Affiliated Hospital and Yuying Children’s Hospital of Wenzhou Medical University, The Second School of Medicine, Wenzhou Medical University, Wenzhou, China

**Keywords:** Subglottic stenosis, Bibliometric analysis, Research trends, VOSviewer, Citespace, Web of Science

## Abstract

**Background:**

Subglottic Stenosis (SGS), with increasing numbers of studies, is the most specific and common clinical type of Laryngotracheal Stenosis (LTS). There is rapid publication turnover with newer management introduced and expanding research field. To our knowledge, there is no bibliometric analysis of SGS yet.

**Methods:**

In August 2022, we performed a thorough search in the Web of Science Core Collection database using the word “subglottic stenosis,” and “SGS.” The 580 articles were arranged based on correlation. The collected articles were then analyzed with an assessment of relevant factors. Meanwhile, we analyzed the top 100 most-cited articles on SGS.

**Results:**

The frequency of publication on SGS has increased substantially over time. The USA has contributed the most articles (*n* = 301). Vanderbilt University published most of the articles among other institutions (*n* = 18). *Laryngoscope* topped the list of journals and has published 89 SGS-related articles. Research hotspots shift from surgical treatment to conservative management.

**Conclusions:**

The SGS-related literature has grown rapidly in recent years. This study represents the first bibliometric analysis of scientific articles on SGS. Areas to improve in SGS research can be identified after this analysis of the most impactful articles on this topic.

## Introduction

Subglottic stenosis (SGS) is a congenital or acquired condition characterized by a narrowing of the upper airway extending from just below the vocal folds to the lower border of the cricoid cartilage [1]. Compared with congenital and acquired, idiopathic subglottic stenosis (iSGS) is a relatively new entity [2, 3]. Idiopathic subglottic stenosis (iSGS), a rare fibrotic disease, is a clinical challenge in terms of etiology, pathogenesis and management [4–6]. The management strategy of SGS depends heavily on the level by Myer-Cotton grading scale. Mild stenoses (grades 1 and 2) treated non-surgically (therapeutic endoscopy) and severe stenoses (grades 3 and 4) usually require surgical intervention (laryngotrachectomy) [1, 7]. Studies have demonstrated that therapeutic endoscopy has fewer complications compared to surgery, however, it has a high recurrence rate and requires repeated interventions [8–10]. Research shows that the extubation rate of T-tube reconstruction after laryngotracheectomy exceeds 95% [11–13].

Currently, research on SGS has expanded rapidly, including etiology, pathogenesis, clinical staging and management patterns. Recently, bibliometric analysis has been widely utilized to quantitatively and qualitatively evaluate the publication trends and hotspots of research, including its authors, journals, institutions, and countries [14–16]. To our knowledge, however, there is no bibliometric analysis of global scientific research on SGS yet.

Here, we performed the first bibliometric study aiming to better understand the research status and publication trends, which may be helpful to determine future direction. Furthermore, we also provide a list of the top 100 most cited articles on SGS between 1968 and 2020.

## Materials and methods

### Search strategy

All data were obtained from the Web of Science Core Collection database. The search was conducted on August 1st, 2022. The search strategy was as follows: Title = (subglottic stenosis OR SGS) AND Document type (article OR review) AND Language = English AND Time span = 1900 to 2022.

### Tools

VOSviewer, CiteSpace and Microsoft Excel 2021 were used to analyze the data. The WoS covers a wide range of publications from different fields [17–19]. VOSviewer and CiteSpace are software tools based on Java which assists with visualization and analysis of bibliometric data. We obtain visualized networks of authors, countries, institutions, co-citation of references, and co-occurrence of keywords.

### Data extraction

The procedure for extracting the bibliometric indicators was carried out by two of the researchers (Z.Y.P and D.Z.Q). Each of the observers recorded the information separately. A third observer collated the records of the first two. This is to demonstrate the accuracy of the information extraction process. In cases where there was no consensus, this third observer (X.J.N) verified each of the indicators and made corrections if needed. A general matrix of the studies was then obtained, which made it possible to analyze the bibliometric indicators. Authors, journals, institutions, countries, and total citations were extracted for data analysis.

## Result

### Publication trend

**Fig. 1:**
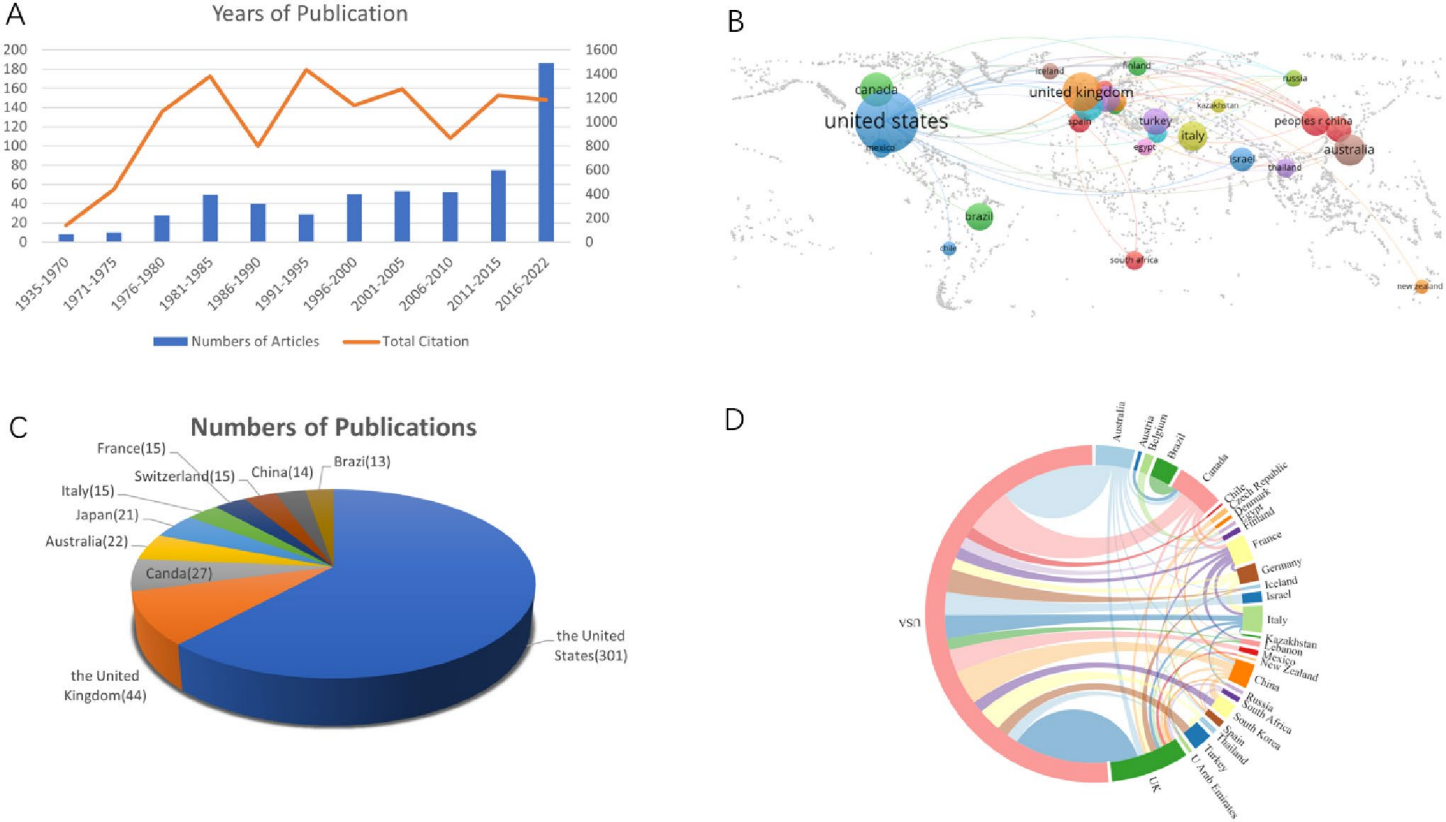
Overview of publications relating to subglottic stenosis (SGS). **(A)** Number of publications and citations from 1935 to 2022. **(B)** Geographic map showing sources of publications. **(C)** Top 10 countries publishing on SGS **(D)** Network visualization map depicting international collaborations investigating SGS

A total of 580 articles were published by 2042 authors from 547 institutions in 44 countries. These articles were published in 138 journals and cited for 13,718 times. Overall, the number of publications increased dramatically, especially after 2015 (Fig. 1A).

### Country distribution

The distribution of the top publication countries is shown in Fig. 1B. Among them, the USA published the largest number of articles (*n* = 301), followed by the UK (*n* = 44), Canda (*n* = 27), and Australia (*n* = 22) (Fig. 1C). That the USA is the leading country by a wide margin in terms of both impact and volume of output is unsurprising in view of the large population size and leading position of the USA in medical research (Fig. 1D).

### Institution distribution

**Fig. 2:**
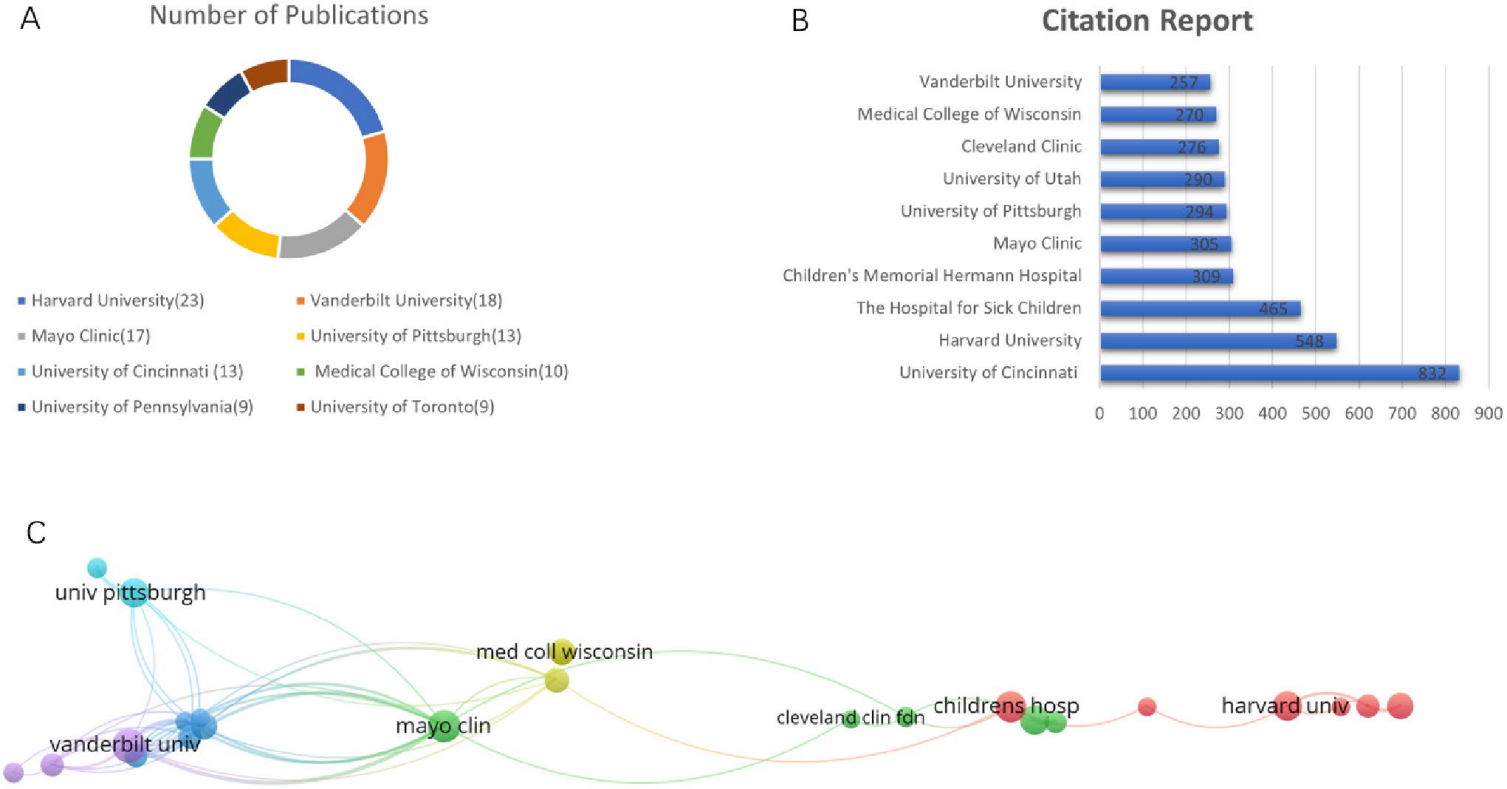
Highest impact institutions publishing on SGS. **(A)** The 8 institutions with the most publications. **(B)** The 10 institutions with the most citations. **(C)** Network visualization map demonstrating institutional collaborations related to SGS

A total of 547 institutions were represented in the published papers. The top 8 institutions were Harvard University (USA; *n* = 23), Vanderbilt University (USA; *n* = 18), Mayo Clinic (USA; *n* = 17), University of Pittsburgh (USA; *n* = 13), University of Cincinnati (USA; *n* = 13), Medical College of Wisconsin (USA; *n* = 10), University of Pennsylvania (USA; *n* = 9), and University of Toronto (Canda; *n* = 9) (Fig. 2A). University of Cincinnati’s publication was cited for most times (832 citations), followed by Harvard University (465 citations) and The Hospital for Sick Children (309 citations) (Fig. 2B).

In terms of collaborative relationships between institutions examined in our network visualization analysis, Vanderbilt University had the highest total link strength (*n* = 67), followed by Mayo Clinic (*n* = 52), Johns Hopkins University (*n* = 41) and University of Utah (*n* = 41). In this analysis, the thickness of the line reflects the frequency of co-authorship collaboration among the institutions (Fig. 2C).

### Journal of publication

The 580 publications were published in 138 academic journals. The top 10 Journals published 62.6% of all publications (Table 1). The top 3 journals were: *Laryngoscope, Annals of otology rhinology and laryngology* and *International journal of pediatric otorhinolaryngology*. *Annals of otology rhinology and laryngology* had the highest number of citations. The Journals with more than 20 of the publications on SGS, mean impact factor (IF) was 2.991.

**Table 1:** Journals publishing most on subglottic stenosis

Rank	Source	Publications	Citations	Mean Citations	Impact Factor
1	Laryngoscope	89	2186	24.56	2.970
2	Annals of Otology Rhinology and Laryngology	88	3352	38.09	1.973
3	International Journal of Pediatric Otorhinolaryngology	56	588	10.50	1.626
4	Otolaryngology-Head and Neck Surgery	37	548	14.811	5.591
5	Journal of Laryngology and Otology	26	239	9.192	2.549
6	Archives of Otolaryngology-Head &Neck Surgery	22	571	25.96	3.236
7	European Archives of Oto-Rhino-Laryngology	15	155	10.33	2.187
8	Pediatric Surgery International	11	37	3.36	2.003
9	Journal of Pediatric Surgery	10	137	13.70	2.327
10	Annals of Thoracic Surgery	9	365	40.56	5.102

### Keywords analysis and research interest

**Fig. 3:**
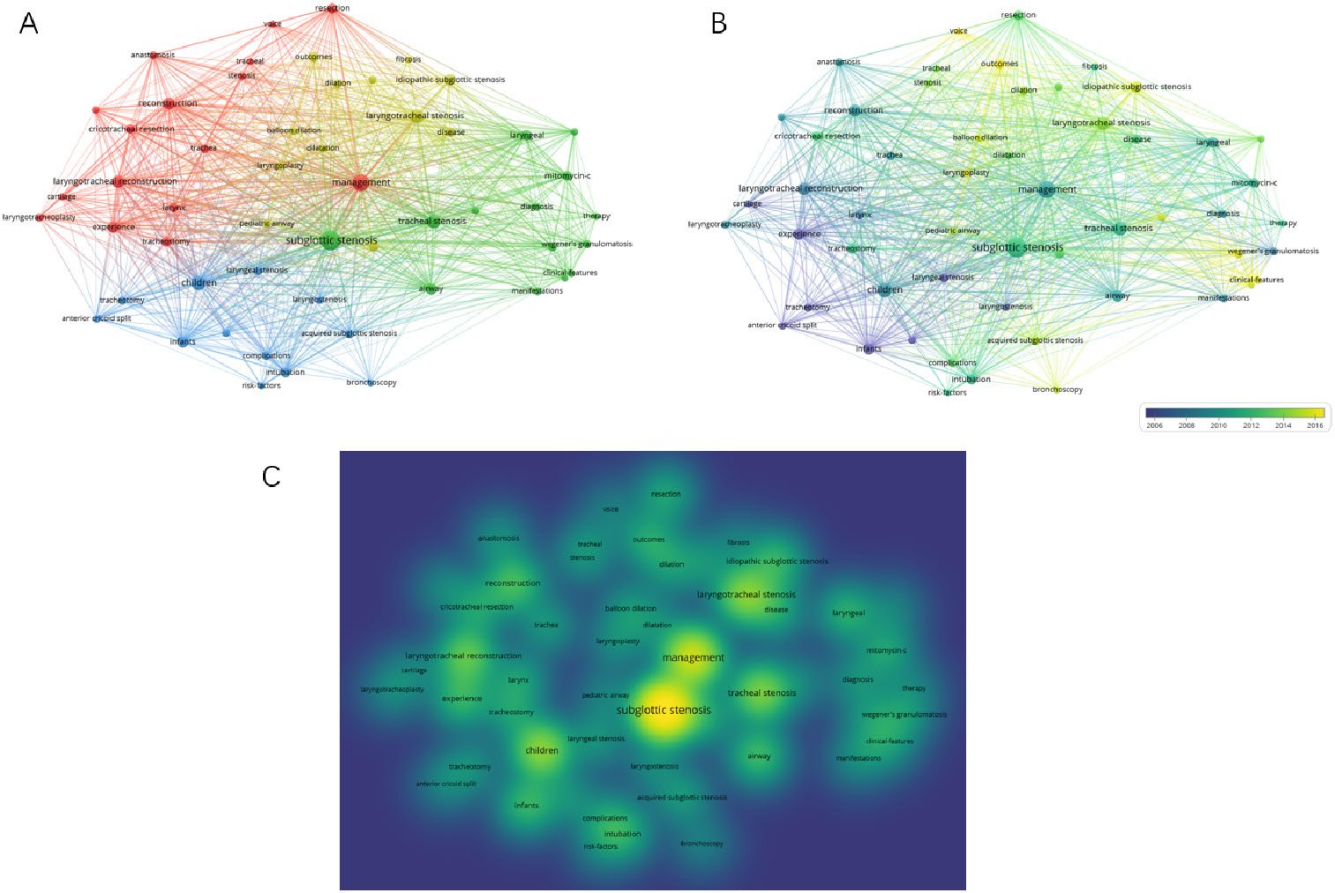
Keyword analysis. **(A)** Network visualization map showing cluster analysis of keywords associated with SGS. **(B)** Network visualization map showing evolution of keyword frequency over time. Colors were assigned according to the average year in which keywords appeared in articles. **(C)** Network visualization map showing density of keywords

Figure 3 shows the co-words network of the most common keywords of the articles published. After setting the minimum number of occurrences of the publication to 10, 54 keywords were obtained. Research areas include susceptible population, classification, management, and outcomes. Figure 3B shows that the keywords change over time, and the color of the keywords varies from year to year. Some keywords frequently appear in each period, such as “subglottic stenosis,” “management,” “tracheal stenosis,” and “laryngotracheal stenosis (Fig. 3C).” In the early days of SGS research, “infants,” “tracheotomy,” “laryngeal stenosis,” “cartilage,” and “anterior cricoid spilt” were the main research hotspots. Notably, “voice,” “balloon dilation,” “idiopathic subglottic stenosis,” and “bronchoscopy,” represented some of the current hotspots.

### The 100 most-cited articles

**Fig. 4:**
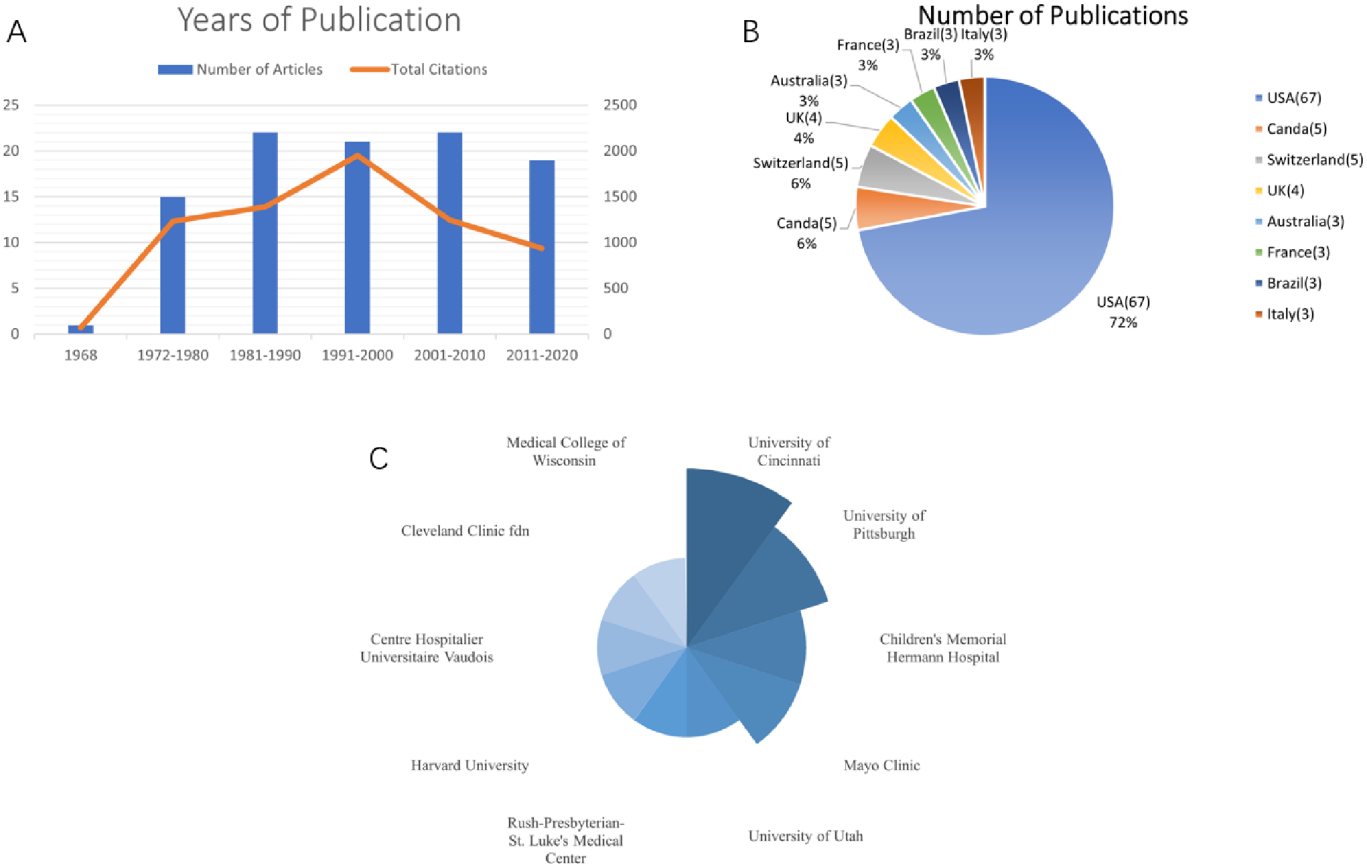
Analysis of the top 100 most-cited publications on SGS. **(A)** Year of publication. **(B)** Distribution of publications by country of origin. **(C)** Institutions with more than three publications

The top 100 most-cited publications on SGS identified in our study were published between 1968 and 2020 (Table 2). The period with most publications was 1981 to 1990 and 2001 to 2010 (*n* = 22 respectively) (Fig. 4A). A total of 16 countries/regions contributed to publications. A visual analysis of the country distribution shows that countries such as the United States (*n* = 67), Canada (*n* = 5), Switzerland (*n* = 5), and the United Kingdom (*n* = 4) are the most notable countries for co-authorship publications, and these countries not only have the highest number of publications but also rank highest in centrality (Fig. 4B).University of Cincinnati contributed 6 publications out of the 100, the highest among the institutions represented, followed by University of Pittsburgh (*n* = 5) (Fig. 4C).

**Table 2:** The top 100 most-cited articles on subglottic stenosis

Rank	Authors	Title	Journal	Citations	Year	Citations/Year
1	Myer, CM	Proposed Grading System for Subglottic Stenosis Based on Endotracheal-Tube Sizes	Annals of Otology Rhinology and Laryngology	503	1994	17.34
2	Little, FB	Effect Of Gastric-Acid on The Pathogenesis of Subglottic Stenosis	Annals of Otology Rhinology and Laryngology	196	1985	5.16
3	Langford, CA	Clinical Features and Therapeutic Management of Subglottic Stenosis in Patients with Wegener’s Granulomatosis	Arthritis and Rheumatism	179	1996	6.63
4	Holinger, PH	Subglottic Stenosis in Infants and Children	Annals of Otology Rhinology and Laryngology	151	1976	3.21
5	Sasaki, CT	Tracheostomy-Related Subglottic Stenosis - Bacteriologic Pathogenesis	Laryngoscope	148	1979	3.36
6	Cotton, R	Management of Subglottic Stenosis in Infancy and Childhood - Review of A Consecutive Series of Cases Managed by Surgical Reconstruction	Annals of Otology Rhinology and Laryngology	140	1978	3.11
7	Fearon, B	Surgical-Correction of Subglottic Stenosis of Larynx in Infants and Children - Progress Report	Annals of Otology Rhinology and Laryngology	123	1974	2.51
8	Grillo, HC	Laryngotracheal Resection and Reconstruction for Subglottic Stenosis	Annals of Thoracic Surgery	123	1992	3.97
9	Walner, DL	Neonatal Subglottic Stenosis - Incidence and Trends	Laryngoscope	122	2001	5.55
10	Grillo, HC	Primary Reconstruction of Airway After Resection of Subglottic Laryngeal and Upper Tracheal Stenosis	Annals of Thoracic Surgery	121	1982	2.95
11	Shapshay, SM	Endoscopic Treatment of Subglottic and Tracheal Stenosis by Radial Laser Incision and Dilation	Annals of Otology Rhinology and Laryngology	115	1987	3.19
12	Lebovics, RS	The Management of Subglottic Stenosis in Patients with Wegener’s Granulomatosis	Laryngoscope	115	1992	3.71
13	Dedo, HH	Endoscopic Laser Repair of Posterior Glottic, Subglottic and Tracheal Stenosis by Division or Micro-Trapdoor Flap	Laryngoscope	114	1984	2.92
14	Gerwat, J	Management of Subglottic Laryngeal Stenosis by Resection and Direct Anastomosis	Laryngoscope	95	1974	1.94
15	Gluth, MB	Subglottic Stenosis Associated with Wegener’s Granulomatosis	Laryngoscope	92	2003	4.60
16	Hoffman, GS	Treatment of Subglottic Stenosis, Due to Wegener’s Granulomatosis, with Intralesional Corticosteroids and Dilation	Journal of Rheumatology	92	2003	4.60
17	Ratner, I	Acquired Subglottic Stenosis in The Very-Low-Birth-Weight Infant	American Journal of Diseases of Children	90	1983	2.25
18	Mehta, AC	Concentric Tracheal and Subglottic Stenosis - Management Using the Nd-Yag Laser for Mucosal Sparing Followed by Gentle Dilatation	Chest	90	1993	3.00
19	Valdez, TA	Idiopathic Subglottic Stenosis Revisited	Annals of Otology Rhinology and Laryngology	90	2002	4.29
20	Monnier, P	Partial Cricoid Resection with Primary Tracheal Anastomosis for Subglottic Stenosis in Infants and Children	Laryngoscope	88	1993	2.93
21	Fearon, B	Surgical Correction of Subglottic Stenosis of Larynx - Preliminary Report of An Experimental Surgical Technique	Annals of Otology Rhinology and Laryngology	86	1972	1.69
22	Maronian, NC	Association of Laryngopharyngeal Reflux Disease and Subglottic Stenosis	Annals of Otology Rhinology and Laryngology	82	2001	3.73
23	Hawkins, DB	Glottic and Subglottic Stenosis from Endotracheal Intubation	Laryngoscope	81	1977	1.76
24	Walner, DL	Gastroesophageal Reflux in Patients with Subglottic Stenosis	Archives of Otolaryngology-Head & Neck Surgery	79	1998	3.16
25	Dedo, HH	Idiopathic Progressive Subglottic Stenosis: Findings and Treatment In 52 Patients	Annals of Otology Rhinology and Laryngology	79	2001	3.59
26	Parkin, JL	Acquired And Congenital Subglottic Stenosis in Infant	Annals Of Otology Rhinology and Laryngology	78	1976	1.66
27	Jindal, JR	Gastroesophageal Reflux Disease as A Likely Cause of Idiopathic Subglottic Stenosis	Annals of Otology Rhinology and Laryngology	77	1994	2.66
28	Cotton, RT	Management of Subglottic Stenosis	Otolaryngologic Clinics of North America	75	2000	3.26
29	Reinisch, L	Inhibition of Subglottic Stenosis with Mitomycin-C in The Canine Model	Annals of Otology Rhinology and Laryngology	74	1999	3.08
30	Maldonado, F	Idiopathic Subglottic Stenosis: An Evolving Therapeutic Algorithm	Laryngoscope	72	2014	8.00
31	Montgomery	Surgical Management of Supraglottic and Subglottic Stenosis	Annals of Otology Rhinology and Laryngology	70	1968	1.27
32	Hseu, AF	Subglottic Stenosis: A Ten-Year Review of Treatment Outcomes	Laryngoscope	69	2014	7.67
33	Jefferson, ND	Subglottic Stenosis	Seminars in Pediatric Surgery	69	2016	9.86
34	Durden, F	Balloon Laryngoplasty as A Primary Treatment for Subglottic Stenosis	Archives of Otolaryngology-Head & Neck Surgery	67	2007	4.19
35	Gelbard, A	Disease Homogeneity and Treatment Heterogeneity in Idiopathic Subglottic Stenosis	Laryngoscope	67	2016	9.57
36	Cotton, RT	Laryngeal Stenosis Following Carbon-Dioxide Laser in Subglottic Hemangioma - Report of 3 Cases	Annals of Otology Rhinology and Laryngology	64	1985	1.68
37	Dankle, SK	Risk-Factors for Neonatal Acquired Subglottic Stenosis	Annals of Otology Rhinology and Laryngology	64	1986	1.73
38	Hartnick, CJ	Surgery for Pediatric Subglottic Stenosis: Disease-Specific Outcomes	Annals of Otology Rhinology and Laryngology	63	2001	2.86
39	Nouraei, SAR	Outcome of A Multimodality Approach to The Management of Idiopathic Subglottic Stenosis	Laryngoscope	63	2013	6.30
40	Contencin, P	Size of Endotracheal-Tube and Neonatal Acquired Subglottic Stenosis	Archives of Otolaryngology-Head & Neck Surgery	61	1993	2.03
41	Monnier, P	Partial Cricotracheal Resection for Severe Pediatric Subglottic Stenosis: Update of The Lausanne Experience	Annals of Otology Rhinology and Laryngology	61	1998	2.44
42	Ward, PH	Composite Hyoid Sternohyoid Muscle Grafts in Humans – Its Use in Reconstruction of Subglottic Stenosis and Anterior Tracheal Wall	Archives of Otolaryngology-Head & Neck Surgery	60	1977	1.30
43	Roediger, FC	Adult Subglottic Stenosis: Management with Laser Incisions and Mitomycin-C	Laryngoscope	59	2008	3.93
44	Blumin, JH	Evidence of Extraesophageal Reflux in Idiopathic Subglottic Stenosis	Laryngoscope	58	2011	4.83
45	Borowiecki, B	Experimental Animal-Model of Subglottic Stenosis	Annals of Otology Rhinology and Laryngology	57	1977	1.24
46	Tucker, GF	Histo-Pathology of Congenital Subglottic Stenosis	Laryngoscope	57	1979	1.30
47	Halstead, LA	Gastroesophageal Reflux: A Critical Factor in Pediatric Subglottic Stenosis	Otolaryngology-Head and Neck Surgery	56	1999	2.33
48	Rahbar, R	Preliminary Results of Intraoperative Mitomycin-C in The Treatment and Prevention of Glottic and Subglottic Stenosis	Journal of Voice	54	2000	2.35
49	Healy, GB	An Experimental-Model for The Endoscopic Correction of Subglottic Stenosis with Clinical-Applications	Laryngoscope	52	1982	1.27
50	Hautefort, C	Balloon Dilation Laryngoplasty for Subglottic Stenosis in Children Eight Years’ Experience	Archives of Otolaryngology-Head & Neck Surgery	52	2012	4.73
51	Choi, SS	Changing Trends in Neonatal Subglottic Stenosis	Otolaryngology-Head and Neck Surgery	51	2000	2.22
52	Jaquet, Y	Partial Cricotracheal Resection for Pediatric Subglottic Stenosis: Long-Term Outcome In 57 Patients	Journal of Thoracic and Cardiovascular Surgery	51	2005	2.83
53	Yamamoto, K	Meta-Analysis of Therapeutic Procedures for Acquired Subglottic Stenosis in Adults	Annals of Thoracic Surgery	49	2011	4.08
54	Manica, D	Association Between Length of Intubation and Subglottic Stenosis in Children	Laryngoscope	49	2013	4.90
55	Koufman, JA	Endoscopic Management of Subglottic Stenosis with The Co2 Surgical Laser	Otolaryngology-Head and Neck Surgery	48	1981	1.14
56	Supance, JS	Acquired Subglottic Stenosis Following Prolonged Endotracheal Intubation - A Canine Model	Archives of Otolaryngology-Head & Neck Surgery	47	1982	1.15
57	Nicklaus, PJ	Evaluation of Neonatal Subglottic Stenosis - A 3-Year Prospective-Study	Laryngoscope	47	1990	1.42
58	Bisson, A	Tracheal Sleeve Resection for Iatrogenic Stenoses (Subglottic Laryngeal and Tracheal)	Journal of Thoracic and Cardiovascular Surgery	47	1992	1.52
59	Dohar, JE	Acquired Subglottic Stenosis - Depth and Not Extent of The Insult Is Key	International Journal of Pediatric Otorhinolaryngology	47	1998	1.88
60	Monnier, P	Cricotracheal Resection for Pediatric Subglottic Stenosis	International Journal of Pediatric Otorhinolaryngology	47	1999	1.96
61	Ciccone, AM	Operative and Non-Operative Treatment of Benign Subglottic Laryngotracheal Stenosis	European Journal of Cardio-Thoracic Surgery	47	2004	2.47
62	Mccaffrey, TV	Management of Subglottic Stenosis in The Adult	Annals of Otology Rhinology and Laryngology	46	1991	1.44
63	Solans-Laque, R	Clinical Features and Therapeutic Management of Subglottic Stenosis in Patients with Wegener’s Granulomatosis	Lupus	46	2008	3.07
64	Zalzal, GH	Rib Cartilage Grafts for The Treatment of Posterior Glottic and Subglottic Stenosis in Children	Annals of Otology Rhinology and Laryngology	45	1988	1.29
65	Maresh, A	A Comparative Analysis of Open Surgery Vs Endoscopic Balloon Dilation for Pediatric Subglottic Stenosis	Jama Otolaryngology-Head & Neck Surgery	45	2014	5.00
66	Finnegan, DA	Hyoid Autograft Repair of Chronic Subglottic Stenosis	Annals of Otology Rhinology and Laryngology	44	1975	0.92
67	Jones, R	Subglottic Stenosis in Newborn Intensive-Care Unit Graduates	American Journal of Diseases of Children	44	1981	1.05
68	Brantigan, CO	Subglottic Stenosis After Cricothyroidotomy	Surgery	44	1982	1.07
69	Marshak, G	Canine Model of Subglottic Stenosis Secondary to Prolonged Endotracheal Intubation	Laryngoscope	43	1982	1.05
70	Macchiarini, P	Partial Cricoidectomy with Primary Thyrotracheal Anastomosis for Postintubation Subglottic Stenosis	Journal Of Thoracic and Cardiovascular Surgery	43	2001	1.95
71	Lang, M	A Systematic Review and Meta-Analysis of Endoscopic Balloon Dilation of Pediatric Subglottic Stenosis	Otolaryngology-Head and Neck Surgery	43	2014	4.78
72	Cotton, RT	Management of Combined Advanced Glottic and Subglottic Stenosis in Infancy and Childhood	Laryngoscope	42	1981	1.00
73	Gelbard, A	Comparative Treatment Outcomes for Patients with Idiopathic Subglottic Stenosis	Jama Otolaryngology-Head & Neck Surgery	42	2020	14.00
74	Park, SS	Idiopathic Subglottic Stenosis	Archives of Otolaryngology-Head & Neck Surgery	41	1995	1.46
75	Quiney, RE	Subglottic Stenosis - A Clinicopathological Study	Clinical Otolaryngology	40	1985	1.05
76	Goode, RL	Long-Term Stenting in Treatment of Subglottic Stenosis	Annals of Otology Rhinology and Laryngology	39	1977	0.85
77	Croft, CB	Therapy of Iatrogenic Subglottic Stenosis - Steroid-Antibiotic Regimen	Laryngoscope	39	1979	0.89
78	Wang, HF	Idiopathic Subglottic Stenosis: Factors Affecting Outcome After Single-Stage Repair	Annals of Thoracic Surgery	39	2015	4.88
79	Gelbard, A	Idiopathic Subglottic Stenosis Is Associated with Activation of The Inflammatory Il-17 A/Il-23 Axis	Laryngoscope	39	2016	5.57
80	Franco, RA	Awake Serial Intralesional Steroid Injections Without Surgery as A Novel Targeted Treatment for Idiopathic Subglottic Stenosis	Laryngoscope	39	2018	7.80
81	Schweiger, C	Incidence Of Post-Intubation Subglottic Stenosis in Children: Prospective Study	Journal Of Laryngology and Otology	38	2013	3.80
82	D’Andrilli, A	Subglottic Tracheal Stenosis	Journal of Thoracic Disease	38	2016	5.43
83	Holinger, LD	Treatment of Severe Subglottic Stenosis Without Tracheotomy - A Preliminary-Report	Annals of Otology Rhinology and Laryngology	37	1982	0.90
84	Singh, T	Subglottic Stenosis Examined as A Fibrotic Response to Airway Injury Characterized by Altered Mucosal Fibroblast Activity	Archives of Otolaryngology-Head & Neck Surgery	37	2010	2.85
85	Giudice, M	Idiopathic Subglottic Stenosis: Management by Endoscopic and Open-Neck Surgery in A Series of 30 Patients	European Archives of Oto-Rhino-Laryngology	36	2003	1.80
86	Damrose, EJ	On The Development of Idiopathic Subglottic Stenosis	Medical Hypotheses	36	2008	2.40
87	George, M	Management of Severe Pediatric Subglottic Stenosis with Glottic Involvement	Journal Of Thoracic and Cardiovascular Surgery	36	2010	2.77
88	Wong, ML	Vascularized Hyoid Interposition for Subglottic and Upper Tracheal Stenosis	Annals of Otology Rhinology and Laryngology	35	1978	0.78
89	Sherman, JM	Decreased Incidence of Subglottic Stenosis Using an Appropriate-Sized Endotracheal-Tube in Neonates	Pediatric Pulmonology	35	1989	1.03
90	Miller, R	Subglottic Stenosis and Down-Syndrome	American Journal of Otolaryngology	35	1990	1.06
91	Poetker, DM	Association of Airway Abnormalities and Risk Factors in 37 Subglottic Stenosis Patients	Otolaryngology-Head and Neck Surgery	35	2006	2.06
92	Terra, RM	Laryngeal Split and Rib Cartilage Interpositional Grafting: Treatment Option for Glottic/Subglottic Stenosis in Adults	Journal of Thoracic and Cardiovascular Surgery	35	2009	2.50
93	Whigham, AS	Outcomes of Balloon Dilation in Pediatric Subglottic Stenosis	Annals of Otology Rhinology and Laryngology	35	2012	3.18
94	Benjamin, B	Idiopathic Subglottic Stenosis: Diagnosis and Endoscopic Laser Treatment	Annals of Otology Rhinology and Laryngology	34	1997	1.31
95	Supance, JS	Antibiotics And Steroids in The Treatment of Acquired Subglottic Stenosis - A Canine Model Study	Annals of Otology Rhinology and Laryngology	33	1983	0.83
96	Lee, KH	Role Of Balloon Dilation in The Management of Adult Idiopathic Subglottic Stenosis	Annals of Otology Rhinology and Laryngology	33	2008	2.20
97	Taylor, SC	Clinical Manifestations and Treatment of Idiopathic and Wegener Granulomatosis-Associated Subglottic Stenosis	Jama Otolaryngology-Head & Neck Surgery	33	2013	3.30
98	Maddalozzo, J	Laryngotracheal Reconstruction for Subglottic Stenosis in Children	Annals of Otology Rhinology and Laryngology	32	1987	0.89
99	Mandour, M	Chronic Subglottic and Tracheal Stenosis: Endoscopic Management Vs. Surgical Reconstruction	European Archives of Oto-Rhino-Laryngology	32	2003	1.60
100	Wolter, NE	Intralesional Corticosteroid Injection and Dilatation Provides Effective Management of Subglottic Stenosis in Wegener’s Granulomatosis	Laryngoscope	32	2010	2.46

Overall, the 100 most cited publications were published in 25 journals. *Annals of Otology Rhinology and Laryngology* was the most popular journal with 30 articles and a total of 2586 citations. It was followed by *Laryngoscope* with 24 articles and 1682 citations. *Archives Of Otolaryngology-Head & Neck Surgery* contributed 8 articles with 444 citations. *Otolaryngology-Head and Neck Surgery*, and *The Journal of Thoracic and Cardiovascular Surgery both* published 5 articles with 444 and 233 citations, respectively (Table 3).

**Table 3:** Top 5 journals publishing the 100 most-cited articles on subglottic stenosis

Rank	Journal	Article	Total Citation	Mean Citation	Impact Factor
1	Annals of Otology Rhinology and Laryngology	30	2586	86.2	1.973
2	Laryngoscope	24	1682	70.1	2.970
3	Archives of Otolaryngology-Head & Neck Surgery	8	444	55.5	1.397
4	Otolaryngology-Head and Neck Surgery	5	233	46.6	5.591
5	The Journal of Thoracic and Cardiovascular Surgery	5	212	42,4	6.439

Regarding authors, Cotton contributed 10 articles, followed by Monnier with 5 articles, Savary, Shapshay and Holinger with 3 articles respectively (Table 4).

**Table 4:** Top 5 authors contributing to the 100 most-cited articles on subglottic stenosis

Author	Articles	First Author	Last Author	Coauthor
Cotton	10	4	1	5
Monnier	5	3	0	2
Savary	4	0	2	2
Shapshay	4	1	0	3
Holinger	4	1	0	3

**Fig. 5:**
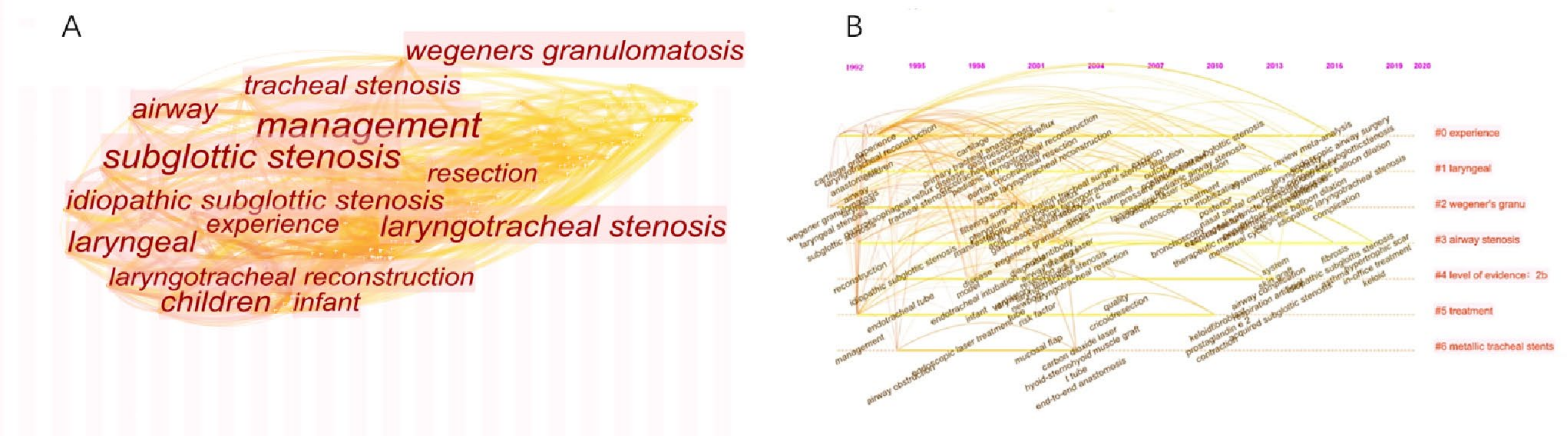
Keyword analysis of the top 100 most-cited publications on SGS. **(A)** Network visualization map showing cluster analysis of keywords associated with SGS. **(B)** Network visualization map showing evolution of keyword frequency over time

The most common topics were “management” (*n* = 23), followed by “subglottic stenosis” (*n* = 20), “laryngotracheal stenosis” (*n* = 13), and “children” (*n* = 13) (Fig. 5A).

The timeline map of keywords can clearly observe the time span of each cluster and the development trend of a specific cluster, explore the time characteristics of research field reflected by each cluster, and thereby verdict the evolution trend of hotspots. According to the Fig. 5B, the longest lasting heat was “airway stenosis,” starting in 1992 and continuing to 2019. These latest keywords are “hypertrophic scar,” “in-office treatment,” and “keloid.” And the shortest duration cluster was “metallic tracheal.”

## Discussion

For newcomers to a field of study, a bibliometric review can provide an invaluable overview of knowledge in the era of big data, allowing them to conduct research more efficiently. Bibliometric analyses are well-established methods for quantifying quality and scholarly impact and have been widely used to assess the developmental characteristics of a given topic.

SGS is the narrowing of the upper airway, which lies between the vocal folds and the lower border of the cricoid cartilage. Studies have shown that up to 12% of all positive cases of infection by novel coronaviruses may require prolonged tracheal intubation for mechanical ventilation or tracheotomy [20]. The most common airway-related complication of this operation is laryngotracheal stenosis, with subglottic stenosis being the most common type [20]. Meanwhile, in the general environment of COVID-19, based on the pathogenesis of SGS and the increase in the number of people undergoing endotracheal intubation, experts are predicting that there will be a large increase in the incidence of post-intubation (PI) SGS [21]. The size of the tracheal tube, duration of intubation, traumatic intubation, presence of infection at the time of intubation, and gastroesophageal reflux are factors thought to play a role in the development of SGS [22, 23]. Research on SGS has now expanded dramatically to include studies of its pathology, Clinical manifestations, natural history, and management. We performed the first bibliometric analysis of SGS literature with the goals of evaluating research trends over time and identifying the most impactful articles.

### Trends in the publication of SGS scientific literature

The United States leads all countries in both total publications and citations, indicating the dominance of the United States in SGS research. In terms of institutional contributions, Harvard University published the most articles with a total of 23, and University of Cincinnati ranked first in total citations (*n* = 832).

In terms of periodicals, the journal analysis can provide important information about high-impact journals. Impact factor (IF), journal citation report categories and total citations are important indicators to measure journal quality. There are nearly 40% of articles focusing on SGS published in *Laryngoscope* (IF = 2.6, Q3), *Annals of otology rhinology and Laryngology* (IF = 1.4, Q3) and *International journal of pediatric otorhinolaryngology* (IF = 1.5, Q4) Table 1. It showed that SGS research results were published mainly in otolaryngology, Head and Neck Surgery, Pediatric Surgery, and Thoracic Surgery.

### Research focuses

The results of keyword analysis showed that “management,” “subglottic stenosis,” “tracheal stenosis,” and “laryngotracheal stenosis” were the centers of keyword clustering, and the research hotspots have gradually changed over time, from initial surgical treatment to conservative treatment. For example, early keywords that appeared more frequently were “tracheotomy,” while more recent keywords appear to be “balloon dilation.”

### The most influential articles

The most cited publication of SGS was “Proposed Grading System for Subglottic Stenosis Based on Endotracheal Tube Sizes” by Myer, CM et al. in 1994 [7]. To harmonize the treatment rules of SGS, they proposed a simple and reproducible System for Subglottic Stenosis based on endotracheal tube sizes. They present a conversion of tube size to the proposed grading scale: grade I up to 50% obstruction, grade II from 51 to 70%, and grade Ill above 70% with any detectable lumen. An airway with no lumen is assigned to grade IV. Patients in grades 1 and 2 are usually treated non-surgically, while surgical treatment is indicated for patients in grades 3 and 4.

“Postintubation Tracheal Stenosis Treatment and results” published by Grillo, HC et al. in 1994 was the second d most-cited article [24]. They found that a patient with tracheal stenosis that had failed to respond to conventional treatment recovered after antacid therapy. They constructed an experimental model of the correlation between gastric acid and airway stenosis consisting of 503 patients with tracheal stenosis. Studies have found that airway narrowing from tracheal intubation can be largely prevented by careful management of the stoma tube.

“Clinical features and therapeutic management of subglottic stenosis in patients with wegener’s granulomatosis” by CA, Langford et al. in 1996 was the third most-cited article [25]. They reviewed 43 cases of patients with SGS and 20 patients treated with endotracheal glucocorticoid injections to determine the clinical features and optimal treatment of subglottic stenosis (SGS) in patients with Wegener’s granulomatosis (WG). Eventually, they found that SGS often occurs independently of other features of active WG and is frequently unresponsive to systemic immunosuppressive therapy.

### Limitations

This study provided bibliometric information related to SGS extracted from Web of Science Core Collection database. Although this analysis was relatively comprehensive and objective, it had several limitations. First, some of influential articles that were not included in this database, so they were excluded from our study. Second, our search criteria were limited to articles in English, we might have missed out some of high-impact articles written in other languages. Third, the date of our retrieval and extraction of data was August 1st,2022. Part of the data correspond to dynamic changes, but the trend of changes will not be extensive.

## Conclusion

This bibliometric analysis showed the increasing trends in published articles related to SGS over the past 10 years. The United States has contributed the most to the SGS literature. *Laryngoscope, Annals of otology rhinology and laryngology* and *International journal of pediatric otorhinolaryngology* are the top three journals with most publications. Conservative treatment has been the focus of recent research. Besides, the 100 most cited papers provide an important reference for future researchers.

## Data Availability

The data used in this study were obtained from web of science.
